# Changes of Meranti, Padauk, and Merbau Wood Lignin during the ThermoWood Process

**DOI:** 10.3390/polym13070993

**Published:** 2021-03-24

**Authors:** Danica Kačíková, Ivan Kubovský, Milan Gaff, František Kačík

**Affiliations:** 1 Faculty of Wood Sciences and Technology, Technical University in Zvolen, T.G. Masaryka 24, 960 01 Zvolen, Slovakia; kacikova@tuzvo.sk (D.K.); kacik@tuzvo.sk (F.K.); 2 Faculty of Forestry and Wood Sciences, Czech University of Life Sciences in Prague, Kamýcká 129, 165 00 Praha 6-Suchdol, Czech Republic; gaff@fld.czu.cz

**Keywords:** meranti, padauk, merbau, thermal treatment, wood lignin

## Abstract

Thermal modification is an environmentally friendly process in which technological properties of wood are modified using thermal energy without adding chemicals, the result of which is a value-added product. Wood samples of three tropical wood species (meranti, padauk, and merbau) were thermally treated according to the ThermoWood process at various temperatures (160, 180, 210 °C) and changes in isolated lignin were evaluated by nitrobenzene oxidation (NBO), Fourier-transform infrared spectroscopy (FTIR), and size exclusion chromatography (SEC). New data on the lignins of the investigated wood species were obtained, e.g., syringyl to guaiacyl ratio values (S/G) were 1.21, 1.70, and 3.09, and molecular weights were approx. 8600, 4300, and 8300 g·mol^−1^ for meranti, padauk, and merbau, respectively. Higher temperatures cause a decrease of methoxyls and an increase in C=O groups. Simultaneous degradation and condensation reactions in lignin occur during thermal treatment, the latter prevailing at higher temperatures.

## 1. Introduction

As a renewable composite material, wood is an ideal building material that is easy to work with and offers advantages such as a high strength-to-weight ratio and lower processing energy. However, dimensional instability is one of its major drawbacks, especially for structural uses. Apart from that, it is also susceptible to fungal degradation and to weathering [1,2,3]. These shortcomings can be minimized by making hydrophilic wood hydrophobic, and thermal treatment is a widely used modification method for this purpose. Some wood products are popular in dark and/or red colour. Wood colour becomes darker and redder with increasing temperature due to formation of chromophores predominantly in lignin [4]. Tropical woods have high commercial value on the market and wood industry due to their good appearance and excellent physical, mechanical, and machinability properties. Despite the immense tree diversity of tropical forests in Brazil, only a few species are well known, explored, and sold in local markets, but many others can provide wood with good properties and high potential for applications in the wood industry [5,6,7]. Nevertheless, it is desirable to improve some of their properties, e.g., durability, stability, pest resistance, and colour uniformity. Thermal treatment appears to be an environmentally friendly and economical technology to improve the properties and colour of wood products. Thermal treatment is suitable for wood since it is non-toxic and does not require the use of chemicals. The thermal modification of wood at temperatures from 180 to 260 °C leads to hemicellulose and lignin degradation. This process changes the chemical composition of the wood and reduces its hygroscopicity. Thus, thermally modified wood tends to be more dimensionally stable than unmodified wood of the same species [8,9,10,11].

One of the main wood components is lignin, the largest source of aromatics on earth, as wood-derived biomass consists of up to 35% of lignin. Lignin is an amorphous cross-linked biopolymer that, in combination with cellulose and hemicelluloses, confers structural stability to plants [12,13]. Many statements about lignin being “energetically utilized” are confessions that come disguised as proud claims, but we still do not know how to utilize lignin on a large scale better than burning it. The vast amounts of technical lignins generated annually by the global pulp and paper industries are still awaiting viable ideas for large-scale and general utilization. In addition, lignin can be used in a broad range of composite materials and serves as a raw material to produce many chemicals [14,15,16]. However, a large amount of lignin is also produced in other wood processing industries, such as in the recycling of wood products, which also includes thermally modified wood, and this work can contribute to its better utilization. Thermally modified wood has been used to improve wood composite properties, e.g., wood–plastic composites, particleboard, etc. [17].

Although heat-treated wood exhibits some improved properties [18], e.g., hydrophobicity, dimensional stability, decay resistance, and darker colour, however, some mechanical properties deteriorate due to the heat treatment [19]. The bending strength properties for keruing (*Dipterocarpus* spp.) and light red meranti (*Shorea* spp.) wood were affected by heat exposure. Both modulus of elasticity (MOE) and modulus of rupture (MOR) values for both thermally treated wood species increased when subjected to temperatures of 150, 170, and 190 °C, except for 210 °C [20]. The lightness of teak and meranti wood was the most affected colour attribute during thermal treatment [21].

African padauk wood is a versatile material, and with declining rosewood resources, the value of this wood species is increasing. Padauk wood is used for making furniture, musical instruments, and for construction purposes. Although many applications are implemented, it is still possible to obtain many new applications of this material [22,23]. Thermal treatment of iroko and padauk wood caused a decrease in its density, colour darkening, and considerable improvement of dimensional stability [24]. Although merbau wood is often used in outdoor applications, its disadvantages include easy leachability of water-soluble substances that stain the surrounding materials. This shortcoming can be solved by thermal treatment [25,26]. Merbau extractives have potential as an impregnating material for low-quality timber to improve its properties [27,28]. The effect of higher temperatures on selected fire safety features of tropical wood shows that the thermal treatment of merbau and meranti wood significantly increased its flammability and accelerated its combustion [29].

Despite intensive research, there is still a lack of data about chemical changes in properties of many tropical woods during their thermal modification. Therefore, the purpose of this study was to provide more detailed information about the effect of thermal modification on lignin changes in meranti, padauk, and merbau wood species.

## 2. Materials and Methods

Light red meranti (*Shorea* spp.), padauk (*Pterocarpus soyauxii* Taub.), and merbau (*Intsia* spp.) wood species with dimensions of 20 × 20 × 300 mm (tangential × radial × longitudinal) were thermally modified at various temperatures [30]. Samples were labelled as 20 (untreated), 160, 180, and 210 (according to the applied temperatures), disintegrated to sawdust, and extracted according to the ASTM Standard Test Method [31]. The acid-insoluble lignin (known as Klason lignin) was determined according to the National Renewable Energy Laboratory (NREL) procedure [32]. Procedures for lignin isolation and conditions of its nitrobenzene oxidation (NBO) were reported recently [33]. The molecular weight distribution (MWD) evaluation of dioxane lignins was performed by the previously described method [33]. Fourier transform infrared spectroscopy (FTIR) of isolated dioxane lignin was performed on a Nicolet iS10 spectrometer with Smart iTR ATR accessory. The spectra were collected in an absorbance mode between 4000 and 650 cm^−1^ by accumulating 32 scans at a resolution of 4 cm^−1^ using diamond crystal. All analyses were performed in four replicates. Statistical analysis was performed by applying a one-way analysis of variance, using the probability theory and Fisher’s F-test.

## 3. Results and Discussion

### 3.1. Changes in Lignin Yields

Lignin is the most stable wood component at thermal treatment. Its yield usually increases during all kinds of biomass pre-treatments accomplished at a low pH and high temperature conditions such as dilute acid pre-treatment, hot water pre-treatment, steam explosion, and at high temperatures. The relative percentage of acid-insoluble lignin (Klason lignin) is higher in the modified material than in that of the untreated ones. This phenomenon is due to formation of pseudo-lignin due to the condensation reactions of degradation products of lignin and polysaccharides [25,34,35]. The results in Table 1 show the different behaviour of meranti, padauk, and merbau wood Klason lignins when subjected to thermal modification. The increase of meranti lignin is relatively high at a temperature of 160 °C, then it decreases unexpectedly. This yield drop can be due to preferential lignin degradation reactions, and it corresponds with the decline of lignin molecular weight determined by size exclusion chromatography (Table 4). In contrast, an increase in lignin was observed after the superheated steam treatment of light red meranti wood [36]. On the other hand, yields of padauk and merbau lignins continuously increase similarly to the increase found during the thermal treatment of various wood species [34,36,37]. We found a higher Klason lignin content (32.42%) with a S/G ratio of 1.21 (Table 2), with differences probably due to different wood species, the location of their origin, etc. Similar results for the S/G ratio were found by Syafii, 2001 [38] for several tropical wood species. He reported that the syringyl to guaiacyl ratio of albizia, gmelina, kapur, and yellow meranti woods are 2.03, 2.02, 1.87, and 1.30, respectively. This means that the lignin structure of the above-mentioned woods is predominated by syringyl units. The content of Klason lignin in yellow meranti was 30.00% [38]. The extraction of lignin from wood by dioxane provides good yields with minimal structural changes, which is why dioxane lignin is often used for structural studies [39,40]. Yields of dioxane lignin increased from 1.7 to 1.8-fold for all three species at a temperature of 210 °C in comparison to the original samples (Table 1). The amount of extracted lignin may depend on the degree of its condensation, but a comparison of the yields of dioxane lignin (Table 1), NBO products (Table 2, Table 3 and Table 4), and its molecular weights (Table 5, Table 6 and Table 7) shows that other factors also influence its yields, e.g., functional groups, crosslinking, etc. These factors result in simultaneous degradation and condensation reactions in lignin during the thermal treatment.

### 3.2. Changes in NBO Products

**Figure 1:**
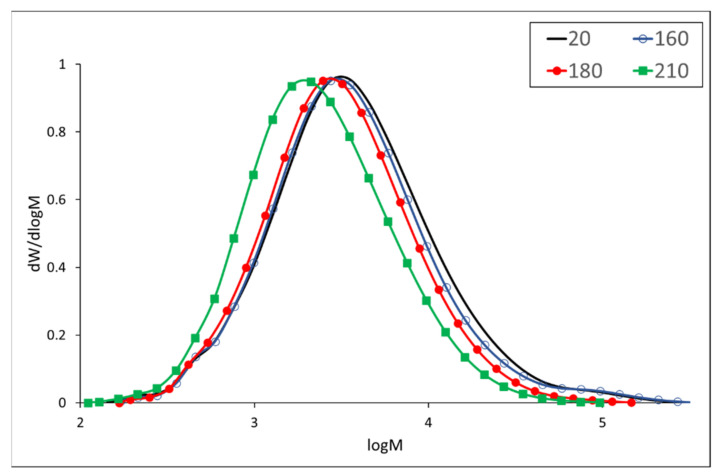
Molecular weight distribution of meranti wood lignins.

The increasing amount of NBO products indicates preferential degradation reactions, which is also confirmed by the drop of molecular weight determined by the SEC analysis (Table 4, Figure 1). The increase of S/G ratio at higher temperatures suggests that G-type lignin is more prone to the condensation reactions [42].

Similarly, to meranti, padauk also has SG lignin, containing more syringyl units compared to guaiacyl ones with an S/G ratio of 1.70, and this ratio slightly increases with the temperature (Table 3). No data of S/G ratio in padauk lignin were found in the literature.

An unexpectedly high S/G ratio was determined in merbau lignin (Table 4), indicating a much lower guaiacyl unit content in comparison to meranti and padauk lignin. The high S/G ratio means that this lignin presents a more open matrix, with a lower degree of C-C bonds at the C5-ring position [43].

### 3.3. Changes of Macromolecular Traits in Lignins

Dioxane lignin isolated from meranti wood before thermal treatment shows typical monomodal molecular weight distribution and a small peak with high molecular weight (Figure 1). Competitive degradation and condensation reactions in lignin can be observed at a temperature of 160 °C, resulting in an increase in the high molecular weight peak (*M*_z_ increases from 41,157 to 48,388 g/mol) and partial degradation of the main peak. At higher temperatures, degradation reactions predominate and both the molecular weights and the polydispersity index decrease, e.g., the value of *M*_w_ drops to half at a temperature of 210 °C (Table 4, Figure 1). It should be noted that no data on the molecular weight of lignin have been found in scientific literature for either meranti or padauk.

**Figure 2:**
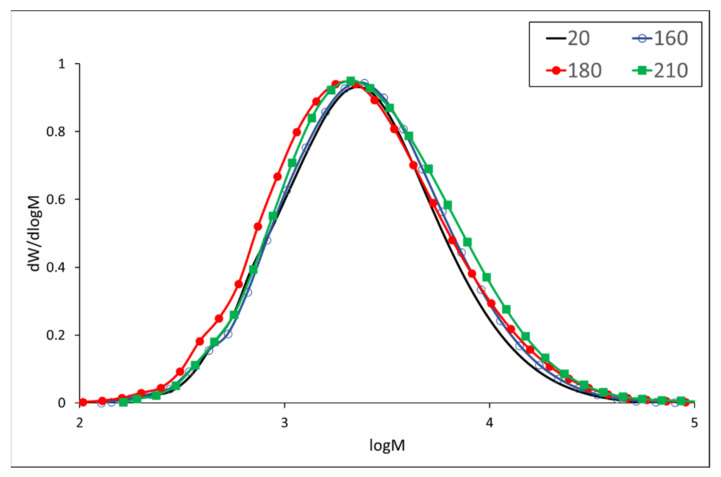
Molecular weight distribution of padauk wood lignin.

Padauk wood lignin reacts to elevated temperatures differently compared to meranti lignin. From a molecular weight of 4265 g/mol in the case of untreated wood, it increases up to a value of 4999 g/mol at a temperature of 210 °C (Table 5). From molecular weight distribution curves, it is evident that both degradation and condensation reactions occur simultaneously, however, the latter are predominant (Figure 2). The SEC analysis similarly revealed concurrent degradation and condensation reactions by heating poplar lignin [42]. The thermal degradation of lignin causes the formation of phenoxy radicals causing a coupling reaction to form new 4−O−5 and 5−5′ linkages, respectively [44,45]. The predominant condensation reactions result in an increase in molecular weight by almost 20% when compared to the unmodified sample. An increase in lignin molecular weight was observed in the thermal treatment of spruce wood up to a temperature of 240 °C [46].

**Figure 3:**
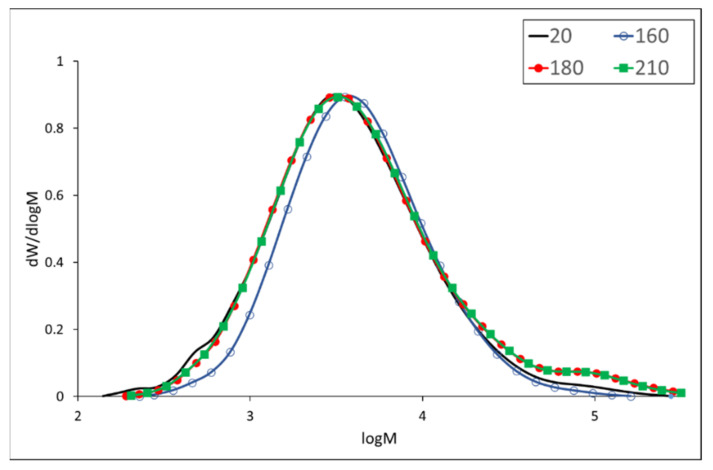
Molecular weight distribution of merbau wood lignin.

The molecular weight of merbau lignin slightly decreases at a temperature of 160 °C (Table 7) and increases dramatically at higher temperatures (by 77%) compared to the untreated sample. Molecular weight distribution curves (Figure 3) show a decrease in both high and low molecular fractions indicating a simultaneous process of degradation and condensation reactions at a lower temperature of treatment. Higher temperatures (180 and 210 °C) predominantly cause the condensation of lignin macromolecules resulting in both higher molecular weights and polydispersity. Similar changes in lignin were found during the thermal treatment of spruce and teak wood, respectively [33,46]. The crosslinking of lignin leading to the increase of molecular weight was observed by [47] during thermoplastic processing of grass lignin with ethylene and vinyl acetate copolymer (EVA).

### 3.4. Changes in FTIR Spectra

**Figure 4:**
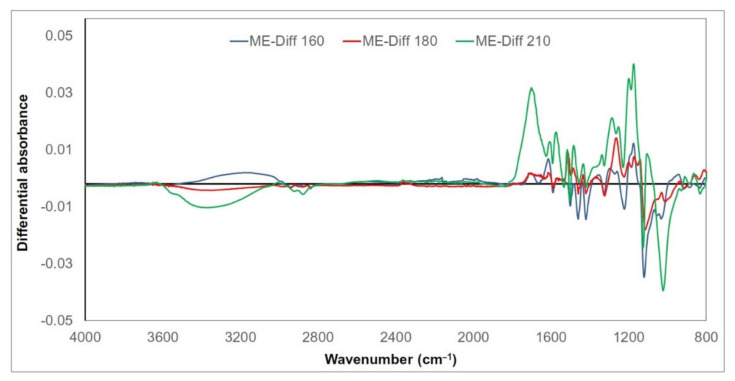
Differential FTIR spectra of thermally treated meranti wood lignin.

**Figure 5:**
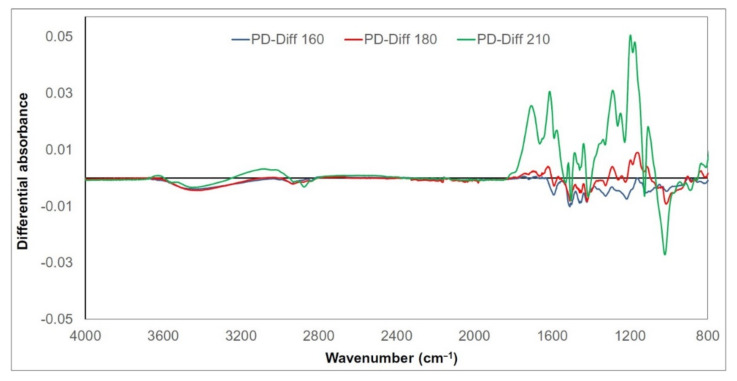
Differential FTIR spectra of thermally treated padauk wood lignin.

**Figure 6:**
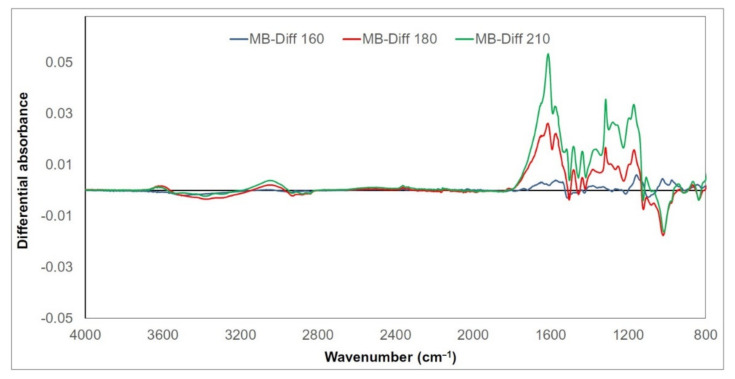
Differential FTIR spectra of thermally treated merbau wood lignin.

Chemical changes in thermally treated wood lignin are expressed as the differential FTIR spectral absorbances of selected related bands (Figure 4,Figure 5 and Figure 6). Their values were obtained as the differences between absorbances after treatment at the given temperature and absorbances of untreated samples (e.g., “ME-Diff 160” means absorbances of meranti lignin treated at 160 °C minus absorbances of untreated meranti lignin). The positive values refer to an increase and the negative values to a decrease in the absorbance. Given the similarity of the trends of the measured absorbances for meranti, padauk, and merbau, the FTIR spectra will be evaluated together (in the case of more significant unequal behavior between them, this will be mentioned). A broad band around 3400 cm^−1^ (OH stretching vibration in alcohols, acids, phenols, and weakly bounded absorbed water in lignin) [22,48] and bands around 2940 and 2840 cm^−1^ (C−H stretching in methyl and methylene groups) [49,50] show only negligible changes in absorbance during the thermal treatment. The absorbance of the band at 1710 cm^−1^ (C=O vibrations of non-conjugated carbonyl groups) increases with the increasing temperature (with a maximum at 210 °C). The increase in intensity may be a result of cleavage of β−O−4 linkage accompanied by the formation of C=O groups formed in lignin during oxidation [51,52]. This trend was observed and explained by several authors as a consequence of increasing the amount of acetyl and carbonyl groups from lignin [53,54]. The absorption intensities near 1600 and 1500 cm^−1^ (C=C stretching vibrations in aromatic structure of lignin) are related to the skeletal vibration of the syringyl and guaiacyl structures in lignin [55,56]. In our case, the band around 1600 cm^−1^ slightly decreased in the meranti and padauk samples treated at lower temperatures (160 and 180 °C) (Figure 1 and Figure 2). On the contrary, it rises at the highest temperature (210 °C), where the increase is higher in padauk (Figure 1 and Figure 2). In the merbau sample, an increase in the band can be observed at each temperature (Figure 3). The band at 1508 cm^−1^ has a different trend in the studied samples. While the meranti and merbau show a gradual increase when subjected to thermal treatment, a permanent decrease can be observed with padauk. An increase in absorbance has been observed by some researchers [57,58], while other authors obtained opposite results [26,52]. The found differences may be due to the different proportions of lignin in the investigated wood species [59]. Absorbance at 1460 cm^−1^ (C−H asymmetric bending in CH_3_ groups in lignin) relating to hemicellulose and methyl groups in lignin [60], shows a variable trend, which is essentially identical as the reported dependence absorbances under thermal treatment at 1593 cm^−1^. In samples treated at lower temperatures (160 and 180 °C), it decreases (Figure 1 and Figure 2). On the contrary, it rises slightly at the highest temperature (210 °C), probably due to the condensation reactions in the lignin structure (Figure 1 and Figure 2). The thermal treatment of lignin causes gradual removal of water and methanol to form conjugated ethylene bonds [61]. A permanent regress at 1420 cm^−1^ (aromatic ring vibration in lignin combined with C−H deformation in carbohydrates) [50] was measured (except for merbau, where the band increased at 210 °C). Decreases in absorbances on bands at 1460 and 1420 cm^−1^ indicate the cleavage of methoxyl groups during thermal treatment leading to gradual demethoxylation [52,62]. The bands at 1325 cm^−1^ (C−O vibration in syringyl plus guaiacyl derivatives is characteristic for condensed structures in lignin) [56,63,64], 1265 and 1215 cm^−1^ (C=O stretching and breathing of guaiacyl ring in lignin) [64,65,66] show a slight decrease at the lowest temperature. This decrease may be due to the cleavage of the ether bond in the lignin structure leading to the elimination of methoxy groups [67]. However, as the temperature rises, the trend reverses and there is a significant increase in these bands (especially in thermal treatment at a temperature of 210 °C). The gradual increase of these bands indicates the development of condensation reactions in lignin [63] (with a significant contribution of guaiacyl). The absorbance value at 1120 cm^−1^ (C−H vibration of syringyl units in lignin) [40] also decreased. The ratio of the relative absorbances of the bands at 1265 and 1120 cm^−1^ shows that a higher temperature induces condensation reactions more easily in guaiacyl units than in syringyl units [68,69]. A decrease of band intensity at 1028 cm^−1^ (C–O stretching) [63] indicate the cleavage of β-alkyl-aryl ether and methoxyl bonds. A similar trend was observed in teak and oak wood [54,57]. These results indicate a significant degradation of the lignin structure, which is most pronounced especially in condensation reactions at the highest temperature value.

### 3.5. Statistical Evaluation Analysis

**Figure 7:**
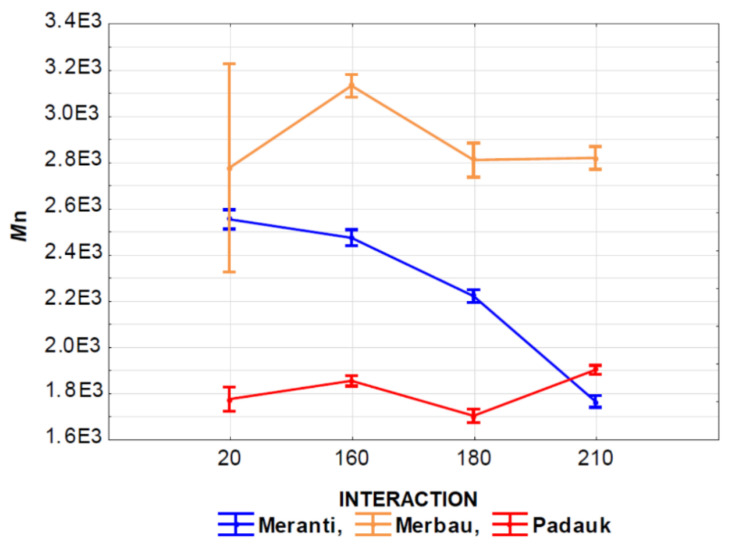
The effect of thermal modification temperature and wood species on number average molecular weight (*M*_n_) values.

**Figure 8:**
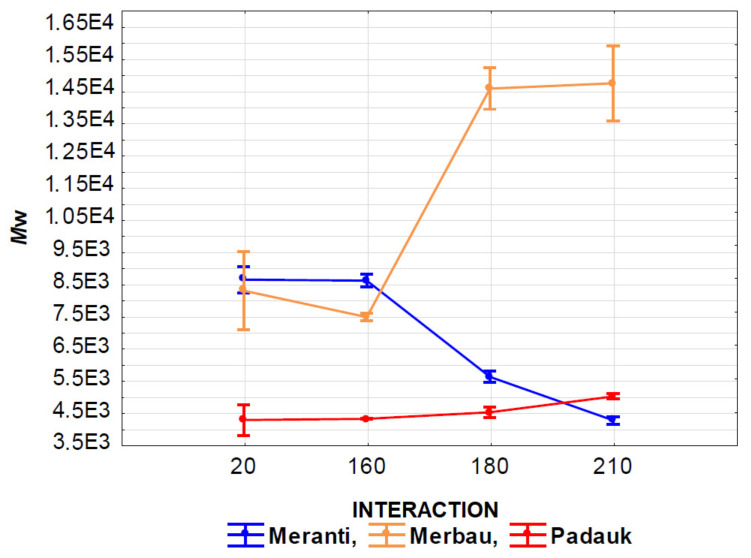
The effect of thermal modification temperature and wood species on weight average molecular weight (*M*_w_) values.

**Figure 9:**
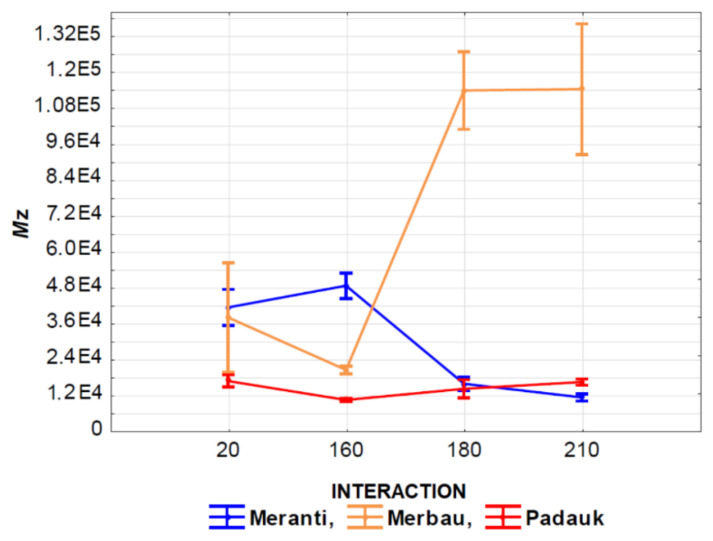
The effect of thermal modification temperature and wood species on Z average molecular weight (*M*_z_) values.

**Figure 10:**
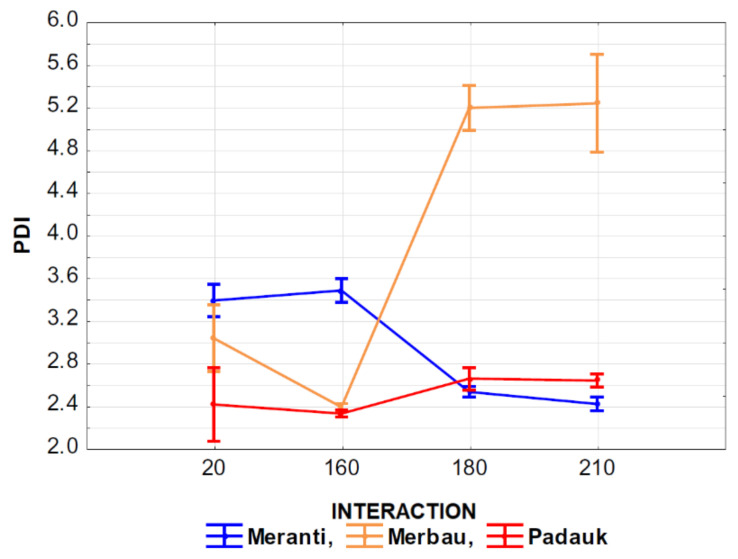
The effect of thermal modification temperature and wood species on polydispersity index.

**Figure 11:**
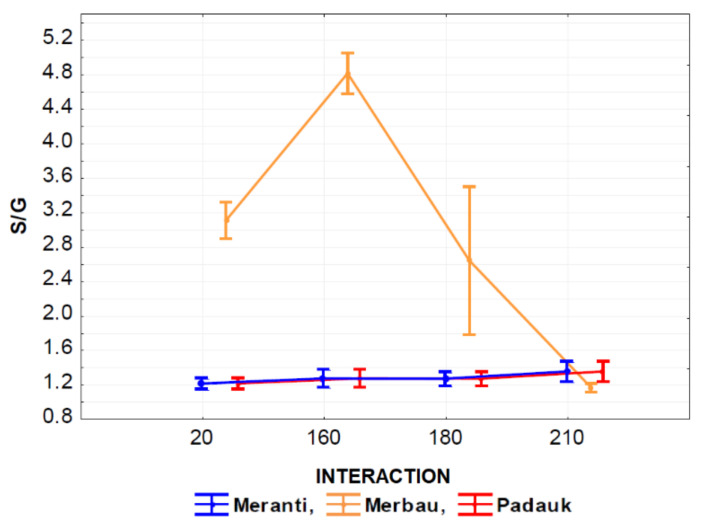
The effect of thermal modification temperature and wood species on syringyl to guaiacyl ratio (S/G) values.

**Figure 12:**
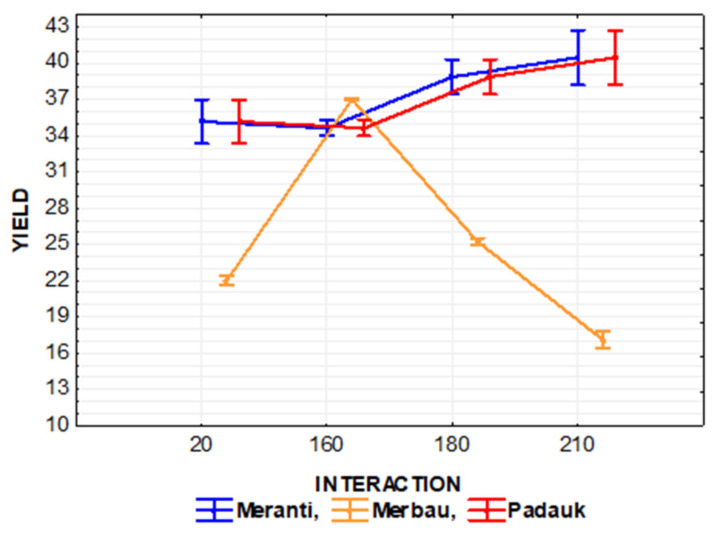
The effect of thermal modification temperature and wood species on YIELD values.

The effect of the interaction of thermal modification temperature (20, 160, 180, and 210 °C) and wood species (meranti, padauk, merbau) was evaluated by applying a one-way analysis of variance, using the probability theory and Fisher’s F-test. Due to the large range of results, we do not present the tables in this article. Based on the values of the level of significance “P” and Fisher’s F-test, these results showed that the effect of the observed interaction (thermal modification temperature and wood species) on the values of *M*_n_, *M*_w_, *M*_z_, S/G, and YIELD has a very significant effect in all monitored cases (Figure 7, Figure 8, Figure 9, Figure 10, Figure 11 and Figure 12).

Figure 7 shows the effect of the interaction of the thermal modification temperature and the wood species on *M*_n_ values. It is clear from the results that the lowest *M*_n_ values were measured in padauk wood, and the highest ones were measured in merbau wood. The effect of temperature on the values of this characteristic was also very significant. In meranti wood, we recorded a significant increase at a temperature of 160 °C, while there was no significant difference between *M*_n_ values measured at 20, 180, and 210 °C. In merbau wood, we can see a significant decrease in *M*_n_ values with an increase in the temperature of thermal modification.

In padauk wood this trend is not so clear-cut. The effect of a temperature of 160 °C increased the values of the examined characteristic, at a temperature of 180 °C there was a significant decrease in the values, and at 210 °C the values of the monitored characteristic increased again.

Figure 8 shows the interaction of the thermal modification temperature and the wood species on *M*_w_ values. The effect of the wood species is similar to the previous case, the lowest *M*_w_ values were measured in padauk wood and the significantly highest values in merbau wood. In the case of padauk wood, the values of the monitored characteristic increased significantly with an increase in the applied temperature. In meranti wood, this effect is opposite, and with an increase in the operating temperature, the values of the observed characteristic of *M*_w_ decrease significantly. An interesting course can be observed in merbau wood, while the effect of 160 °C cannot be considered significant compared to thermally untreated wood. Temperatures of 180 and 210 °C resulted in a very significant increase in the value of the observed characteristic *M*_w_.

Figure 9 shows the effect of the interaction of the temperature of thermal modification and wood species on *M*_z_ values. It is evident from the value in the graph that the lowest values of the observed characteristic were measured in padauk wood again, and the highest values of the monitored characteristic were measured in merbau wood. In padauk wood, a decrease in the values of the observed characteristic *M*_z_ can be observed at a temperature of 160 °C in comparison with the untreated wood. With higher temperatures (180 and 210 °C), we achieved an increase in the values of the observed characteristics. In meranti wood, the effect was the opposite. At 160 °C the values of the monitored characteristic increased insignificantly, but at temperatures of 180 and 210 °C there was a very significant decrease in the values of the monitored characteristic. The effect of the interaction of the thermal modification temperature and the wood species on the values of the observed characteristic PDI is very similar as in the previous case (Figure 10).

The interaction of thermal modification temperature and wood species was shown to have an insignificant effect on the monitoring of this interaction on S/G values measured in padauk and meranti wood (Figure 11). While the effect of the monitored interaction had a very significant effect on the values measured in merbau wood, the values measured in this wood were significantly higher than in the case of meranti and padauk wood. We can also see a significant increase in the values of the monitored characteristic at 160 °C, accompanied by a significant decrease in these values at higher temperatures (180 and 210 °C).

YIELD values measured in padauk and meranti wood do not differ. In both woods, an insignificant increase in the values of the monitored characteristic can be observed due to the thermal modification temperature. In contrast, the YIELD values measured in merbau wood decrease significantly under the effect of thermal modification temperature (Figure 12).

**Figure 13:**
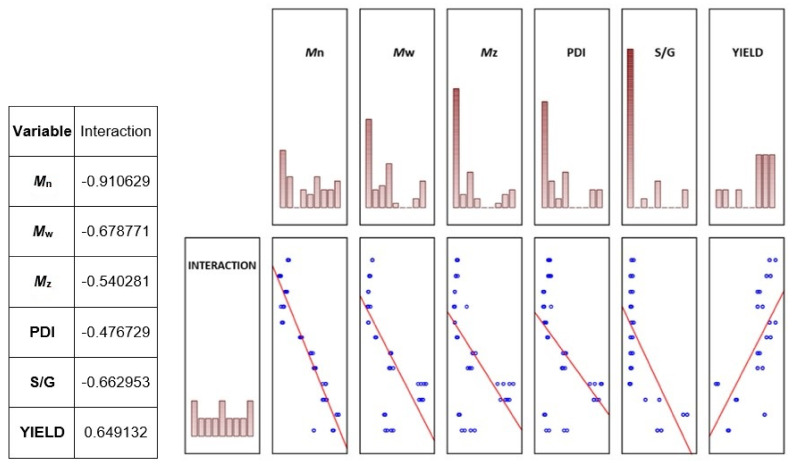
Correlations of the interaction of thermal modification temperature and wood species with the values of the monitored characteristics *M*_n_, *M*_w_, *M*_z_, S/G, and YIELD evaluating the mutual connection between the monitored relationships.

Figure 13 shows the relationship between the observed interaction (thermal modification temperature and wood species) and its influence on the monitored characteristics *M*_n_, *M*_w_, *M*_z_, S/G, and YIELD. It is clear from the values of the correlation coefficient and the data in Figure 13 that the effect of the observed interaction has a significant correlation in all monitored cases, and each of the monitored characteristics has a declining trend as a result of the interaction.

## 4. Conclusions

Meranti, padauk, and merbau wood samples were thermally treated according to the ThermoWood process at different temperatures (160, 180, 210 °C), lignin was extracted by dioxane and its changes were evaluated by chemical, chromatographic, and spectroscopic analyses. Different changes in all the examined lignins were observed. However, simultaneous degradation and condensation reactions take place during the thermal modification of wood samples. In meranti lignin, the loss of molecular weight occurs, in padauk only the negligible increased, but merbau lignin dramatically condensed at the temperature above 180 °C. All the examined wood species are characterized by an SG type of lignin, with more syringyl units compared to guaiacyl, and with a minor content of hydroxyphenyl-units. Nitrobenzene oxidation (NBO) analyses show a similar S/G ratio and yield products in meranti and padauk lignins, the higher S/G ratio and products yield at the temperature of 160 °C was found in merbau lignin, then these values rapidly decreased. Infrared spectra also indicated significant changes in lignin structure, which is most pronounced especially in condensation reactions at the highest temperature value. The knowledge about the changes of lignin structure during the heat treatment could be useful in the ThermoWood treatment, recycling of thermally treated wood or in its processing at the end of the life cycle.

## Figures and Tables

**Table 1:** Klason (KL) and dioxane lignin (DL) yields from untreated and thermally treated meranti, padauk, and merbau (mean ± SD, % odw).

Product	20 °C	160 °C	180 °C	210 °C
Meranti-KL	32.42 ± 0.08	36.67 ± 0.10	36.29 ± 0.09	35.33 ± 0.13
Padauk-KL	33.77 ± 0.10	34.84 ± 0.04	35.53 ± 0.03	39.73 ± 0.09
Merbau-KL	33.75 ± 0.23	33.23 ± 0.07	35.75 ± 0.20	44.61 ± 0.23
Meranti-DL	7.57 ± 0.10	6.79 ± 0.11	7.32 ± 0.06	13.54 ± 0.11
Padauk-DL	9.02 ± 0.16	10.72 ± 0.41	14.20 ± 0.25	16.62 ± 0.31
Merbau-DL	9.01 ± 0.11	7.97 ± 0.12	13.69 ± 0.28	15.37 ± 0.11

The lignin content in meranti (*Shorea almon*) is 23.10% with the ratio of syringyl to guaiacyl (S/G) 0.94 [41].

**Table 2:** Phenolic aldehydes and acids from nitrobenzene oxidation of meranti dioxane lignins (mean ± SD, %).

Product	20 °C	160 °C	180 °C	210 °C
*p*-Hydroxybenzoic acid	0.04 ± 0.00	0.05 ± 0.00	0.04 ± 0.01	0.03 ± 0.01
*p*-Hydroxybenzaldehyde	1.93 ± 0.12	1.44 ± 0.41	1.93 ± 0.12	0.98 ± 0.16
Vanillic acid	0.33 ± 0.00	0.46 ± 0.03	0.65 ± 0.07	0.16 ± 0.03
Vanilline	14.71 ± 0.25	14.19 ± 0.45	15.67 ± 0.09	16.66 ± 0.20
Syringic acid	0.39 ± 0.03	0.38 ± 0.08	0.32 ± 0.00	0.42 ± 0.02
Syringaldehyde	17.77 ± 1.01	18.13 ±0.50	20.26 ± 1.01	22.21 ± 1.76
Total yield on DL	35.17 ± 1.41	34.64 ± 0.52	38.87 ± 1.10	40.47 ± 1.75
S/G ratio	1.21 ± 0.05	1.26 ± 0.08	1.26 ± 0.06	1.35 ± 0.09

**Table 3:** Phenolic aldehydes and acids from nitrobenzene oxidation of padauk dioxane lignins (mean ± SD, %).

Product	20 °C	160 °C	180 °C	210 °C
*p*-Hydroxybenzoic acid	0.04 ± 0.01	0.05 ± 0.00	0.04 ± 0.00	0.05 ± 0.00
*p*-Hydroxybenzaldehyde	3.19 ± 0.12	3.17 ± 0.33	3.08 ± 0.04	3.17 ± 0.16
Vanillic acid	1.09 ± 0.05	1.04 ± 0.16	0.98 ± 0.01	0.92 ± 0.05
Vanilline	7.61 ± 0.07	8.67 ± 0.22	7.96 ± 2.52	3.89 ± 0.07
Syringic acid	1.49 ± 0.06	1.43 ± 0.20	1.41 ± 0.02	1.47 ± 0.09
Syringaldehyde	13.33 ± 1.26	15.28 ± 1.51	14.22 ± 0.50	7.11 ± 0.00
Total yield on DL	26.76 ± 1.56	29.64 ± 1.97	27.68 ± 2.96	16.61 ± 0.24
S/G ratio	1.70 ± 0.13	1.72 ± 0.19	1.75 ± 0.45	1.78 ± 0.02

**Table 4:** Phenolic aldehydes and acids from nitrobenzene oxidation of merbau dioxane lignins (mean ± SD, %).

Product	20 °C	160 °C	180 °C	210 °C
*p*-Hydroxybenzoic acid	0.02 ± 0.00	0.04 ± 0.00	0.02 ± 0.00	0.01 ± 0.00
*p*-Hydroxybenzaldehyde	2.86 ± 0.02	3.26 ±0.16	3.46 ± 0.44	3.23 ± 0.12
Vanillic acid	3.49 ± 0.19	3.57 ± 0.17	4.49 ± 0.98	6.13 ± 0.19
Vanilline	1.18 ± 0.08	2.24 ± 0.02	1.59 ± 0.05	0.30 ± 0.02
Syringic acid	1.24 ± 0.05	1.41 ± 0.11	1.52 ± 0.29	1.44 ± 0.03
Syringaldehyde	13.19 ± 0.46	26.50 ± 0.48	14.18 ± 0.85	6.00 ± 0.45
Total yield on DL	21.98 ± 0.30	37.02 ± 0.05	25.26 ± 0.18	17.11 ±0.59
S/G ratio	3.09 ± 0.16	4.80 ± 0.18	2.58 ± 0.66	1.16 ± 0.04

**Table 5:** Molecular weights and polydispersity index of lignin from meranti wood (mean ± SD).

Temperature (°C)	*M*_w_ (g/mol)	*M*_n_ (g/mol)	*M*_z_ (g/mol)	PDI
20	8627 ± 256	2549 ± 26	41,157 ± 3769	3.38 ± 0.09
160	8595 ± 122	2470 ± 22	48,388 ± 2696	3.48 ± 0.07
180	5607 ± 109	2217 ± 17	15,651 ± 1432	2.53 ± 0.03
210	4253 ± 75	1761 ± 16	11,071 ± 763	2.42 ± 0.04

Note: *M*_w_: Weight average molecular weight (MW); *M*_n_: Number average MW; *M*_z_: Z average MW; PDI (polydispersity index): *M*_w_/*M*_n_.

**Table 6:** Molecular weights and polydispersity index of lignin from padauk wood (mean ± SD).

Temperature (°C)	*M*_w_	*M*_n_	*M*_z_	PDI
20	4265 ± 303	1771 ± 32	10,341 ± 529	2.41 ± 0.22
160	4301 ± 103	1850 ± 14	10,237 ± 279	2.32 ± 0.02
180	4504 ± 105	1698 ± 18	12,972 ± 494	2.65 ± 0.07
210	4999 ± 48	1898 ± 12	16,264 ± 669	2.63 ± 0.04

Note: See Table 5 for symbols.

**Table 7:** Molecular weights and polydispersity index of lignin from merbau wood (mean ± SD).

Temperature (°C)	*M*_w_	*M*_n_	*M*_z_	PDI
20	8284 ± 757	2772 ± 283	37,826 ± 1526	2.99 ± 0.17
160	7461 ± 72	3128 ± 31	20,268 ± 787	2.39 ± 0.02
180	14,567 ± 107	2806 ± 46	113,672 ± 8141	5.19 ± 0.13
210	14,728 ± 729	2815 ± 31	114,080 ± 13,700	5.23 ± 0.29

Note: See Table 5 for symbols.

## Data Availability

Data sharing not applicable.
